# Correction: Separating Timing, Movement Conditions and Individual Differences in the Analysis of Human Movement

**DOI:** 10.1371/journal.pcbi.1005254

**Published:** 2016-12-06

**Authors:** The PLOS Computational Biology Staff

S1 Text is provided as a .TEX file only. Please view the PDF copy of S1 Text here.

## Supporting Information

S1 Text Supporting information contains a primer on model building using the proposed framework, an example analysis with accompanying R code, a simulation study that evaluates the proposed algorithm for maximum likelihood estimation, and the different parameter grids used for cross-validation in the paper. (PDF)
