# Supplementary figures and images for: Separating Timing, Movement Conditions and Individual Differences in the Analysis of Human Movement
Source: PLoS Comput Biol. 2016 Sep 22;12(9):e1005092. doi: 10.1371/journal.pcbi.1005092 (PMC5033575; doi:10.1371/journal.pcbi.1005092)

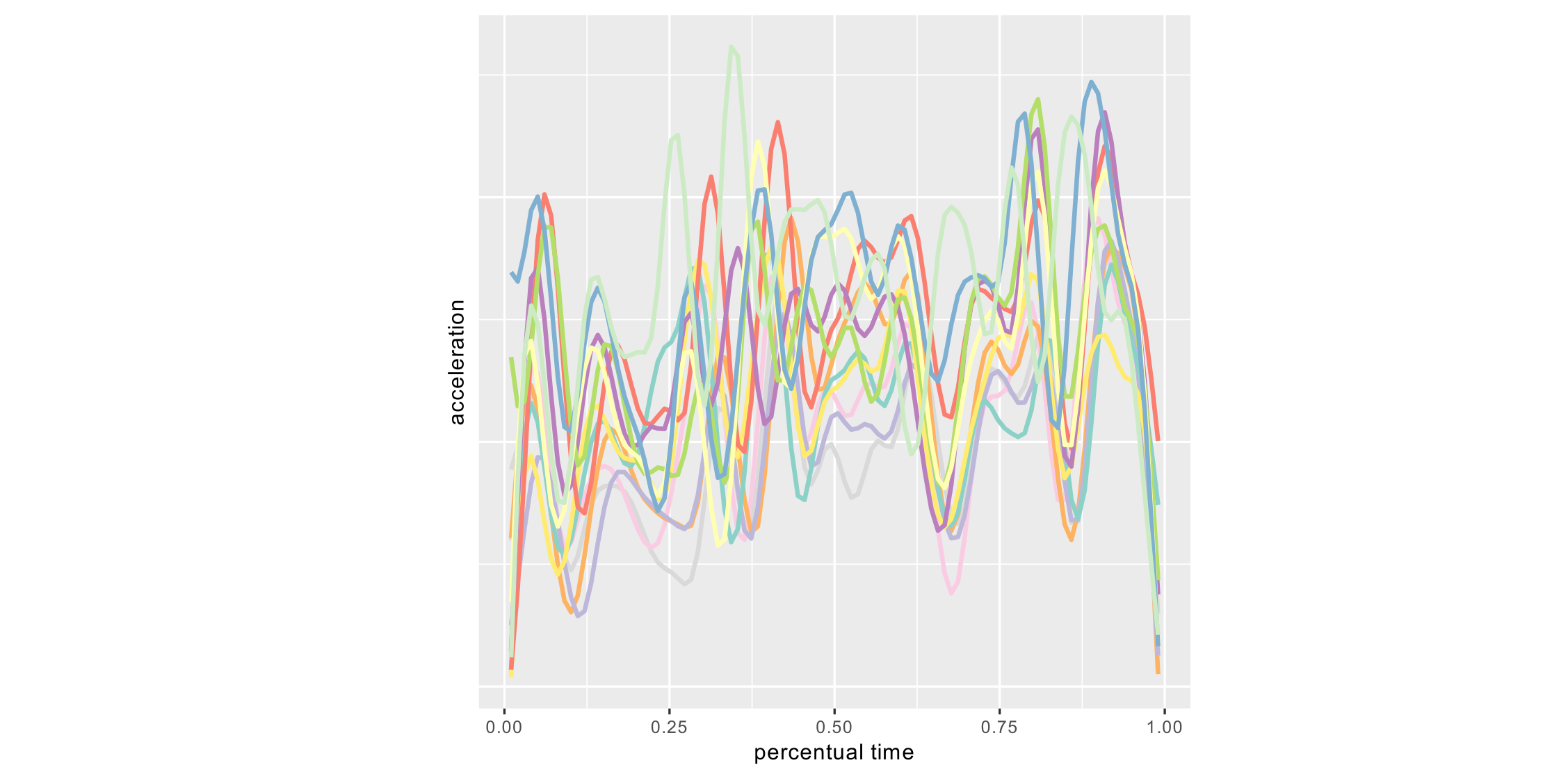

Supplement: S1 Fig — (TIFF) [file pcbi.1005092.s002.tiff]

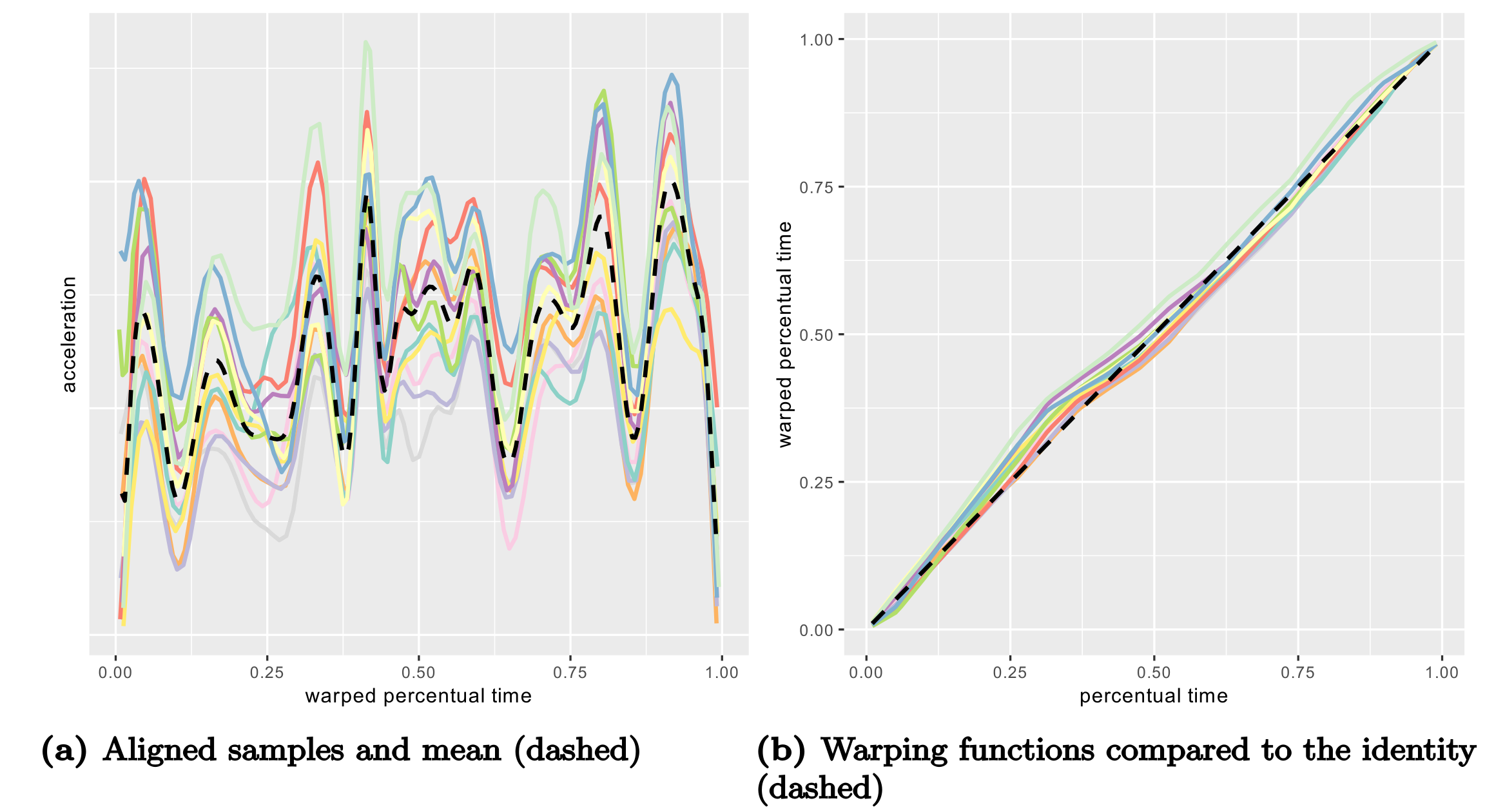

Supplement: S2 Fig — (TIFF) [file pcbi.1005092.s003.tiff]

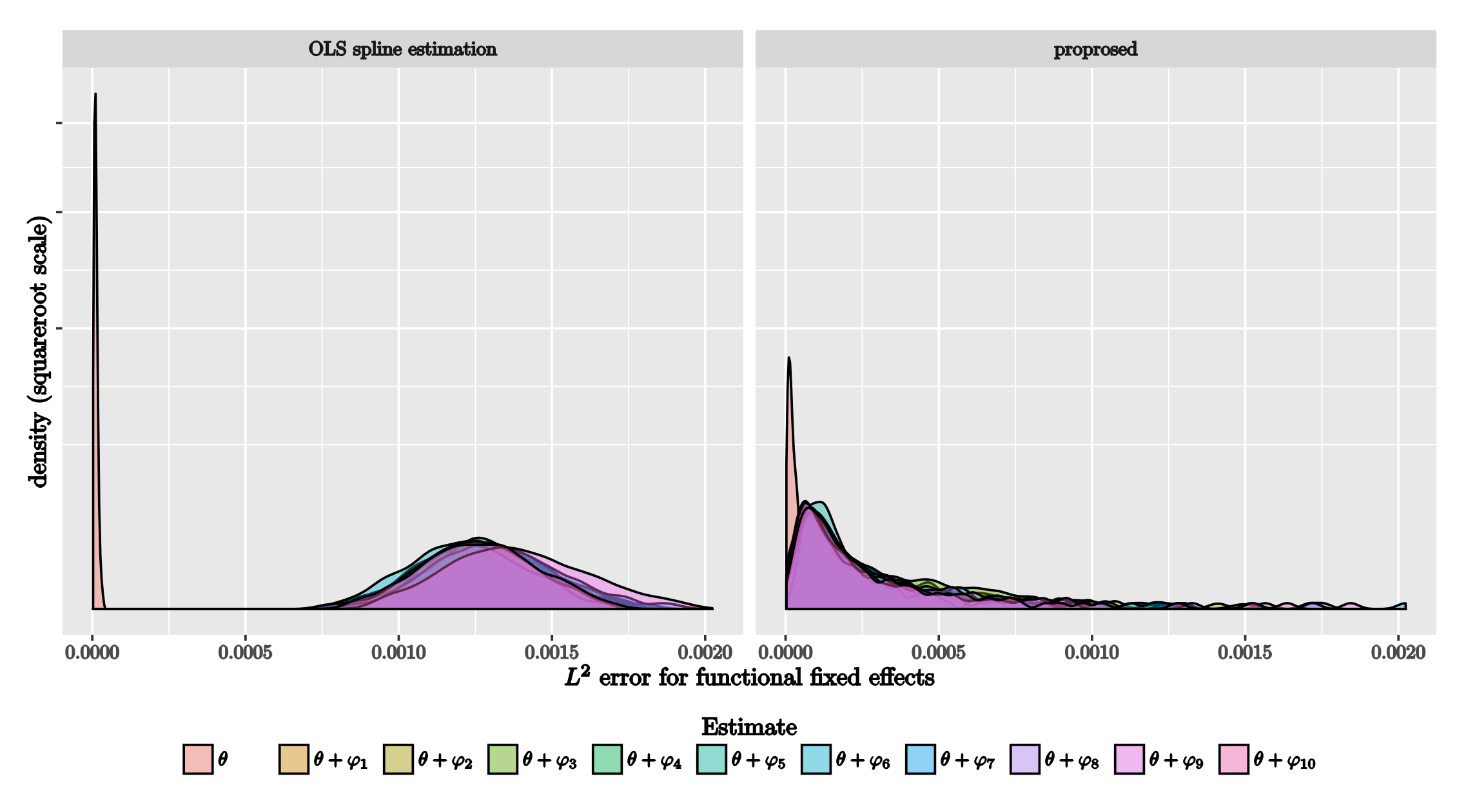

Supplement: S3 Fig — The left panel shows results for ordinary least square (OLS) estimation and the right panel shows the results for the proposed model and estimation algorithm. Both models were fitted using the correctly specified spline model for the mean. Note that the density is displayed on squareroot scale. (TIFF) [file pcbi.1005092.s004.tiff]

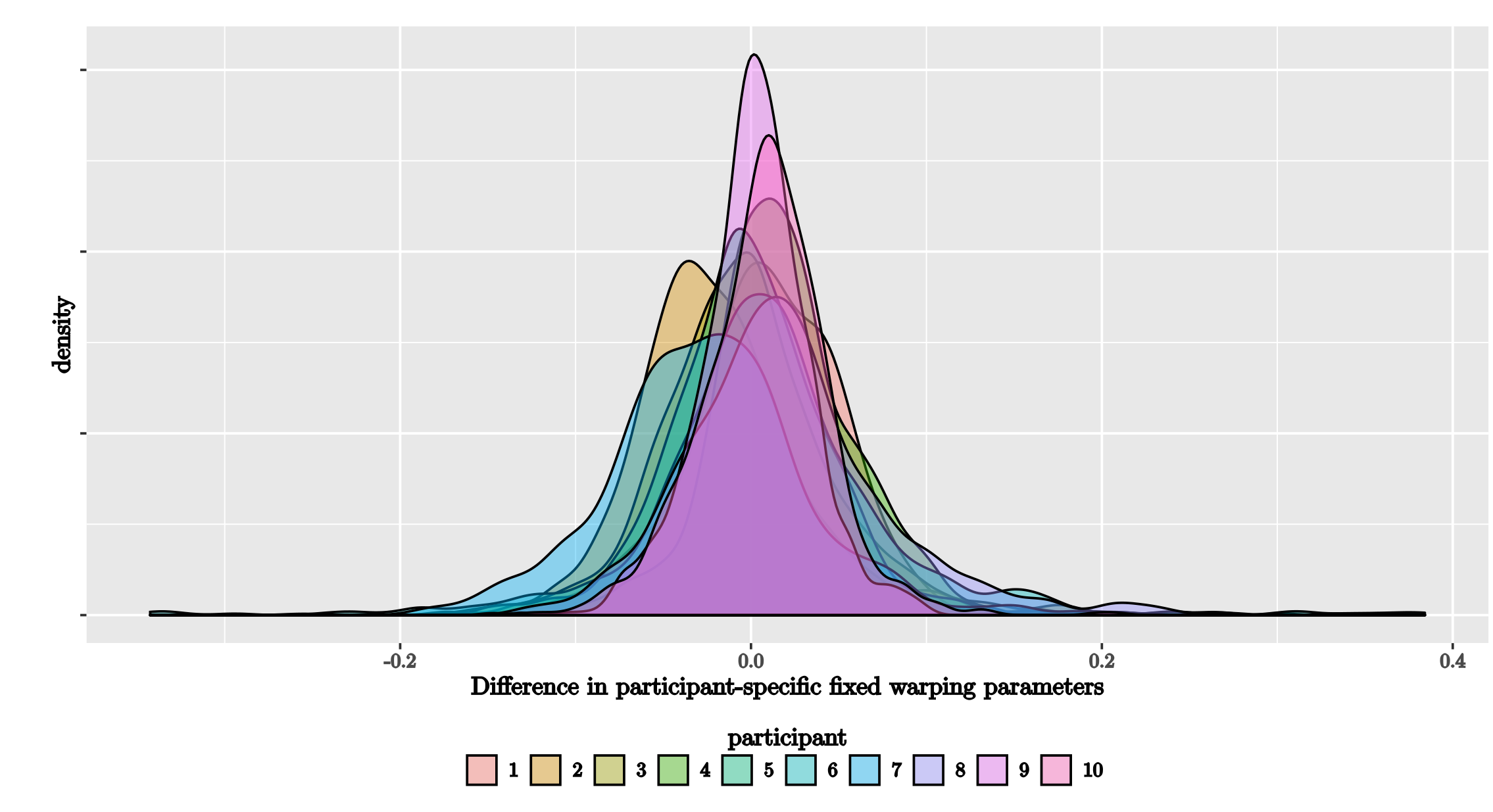

Supplement: S4 Fig — (TIFF) [file pcbi.1005092.s005.tiff]

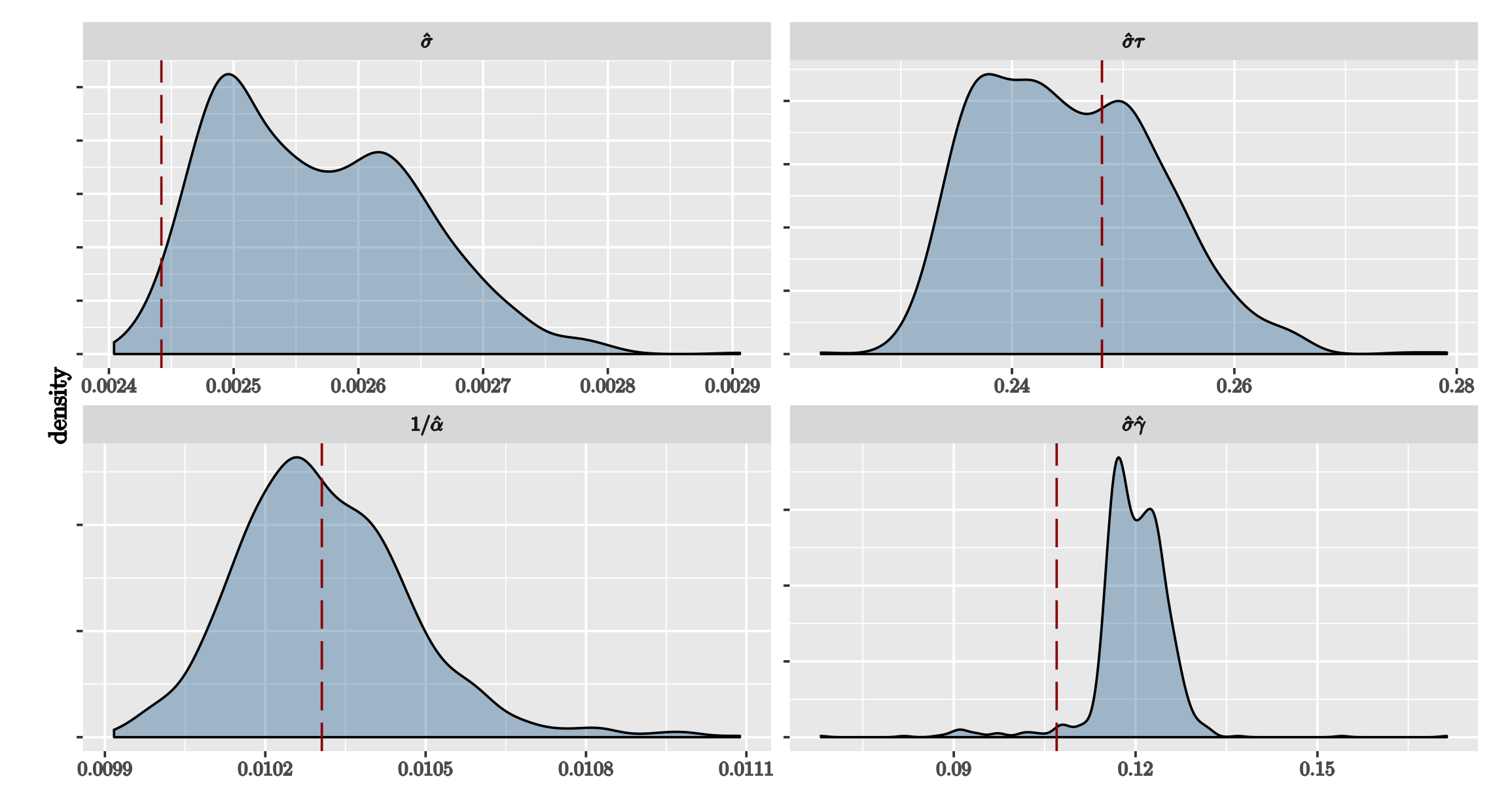

Supplement: S5 Fig — Dashed red lines indicate the true values of the parameters. (TIFF) [file pcbi.1005092.s006.tiff]
