# Supplementary material for: Correction: Separating Timing, Movement Conditions and Individual Differences in the Analysis of Human Movement
Source: PLoS Comput Biol. 2016 Dec 6;12(12):e1005254. doi: 10.1371/journal.pcbi.1005254 (PMC5140050; doi:10.1371/journal.pcbi.1005254)
Supplement: S1 Text — (PDF) [file pcbi.1005254.s001.pdf]

Separating timing, movement conditions and individual differences in the analysis of human movement

Lars Lau Raket[1,*], Britta Grimme[2], Gregor Schöner[2], Christian Igel[3], Bo Markussen[1]

1 Department of Mathematical Sciences, University of Copenhagen, Copenhagen, Denmark
2 Institut für Neuroinformatik, Ruhr-Universität Bochum, Bochum, Germany
3 Department of Computer Science, University of Copenhagen, Copenhagen, Denmark

*** larslau@math.ku.dk**

Supporting Information

A primer on model building for movement data

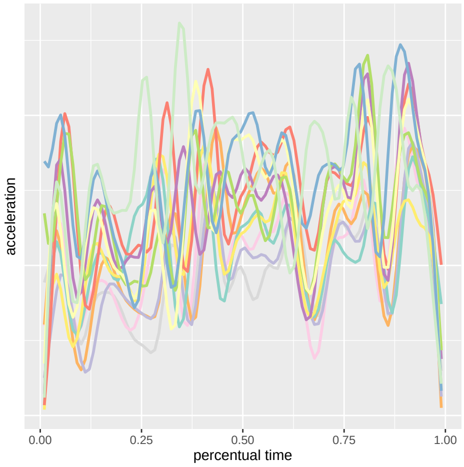

Figure S1. Twelve acceleration functions corresponding to repetitions of a signature being written.

The two major types of variation in movement data are path variation (amplitude) and movement timing variation (time warping). From a statistical modeling perspective, it is natural to model these effects as realizations of random processes across repetitions of the task at hand, since this allows a data driven regularization of the predictions of these effects. We consider the class of models on the form

$$y_i(t_k) = \theta(v(t_k, \boldsymbol{w}_i)) + x_i(t_k) + \varepsilon_{ik} \tag{1}$$

where θ is the mean profile, v is a warping function that depends on the random warping parameters $\boldsymbol{w}_i \in \mathbb{R}^{n_w}$ that are assumed to be independent across i and multivariate zero-mean Gaussian with covariance matrix $\sigma^2 C$, the x_i terms are

independent zero-mean Gaussian process with covariance function $\sigma^2\mathcal{S}$, and the ε_{ik}-terms are independent zero-mean Gaussian noise with variance σ^2.

To use model (1) we need to choose the type of mean function θ, the type of warping function v as well as the covariance structures for $\boldsymbol{w}_i$ and x_i. Below is a list of considerations of how to do these model choices based on the experiment at hand.

θ: A good allround choice is to model θ as a B-spline using a functional basis. For periodic movement sequences, a Fourier basis may sometimes be preferred. To choose the number of basis functions to use we need to consider the data at hand. For experiments with dense sampling and a clear systematic pattern in the trajectories the number of basis functions should be just be sufficiently high to model the mean pattern. If on the other hand the trajectories are sparsely observed in time or the common pattern is very unclear, one should choose a small number of basis functions to avoid local overfitting.

v: The behavior of the warping function should be driven by the random variables $\boldsymbol{w}_i$. We will consider warping functions where $\boldsymbol{w}_i$ models disparities from the identity mapping (corresponding to no warp), and v is an interpolating function of these random disparities at a set of specified anchor points $\boldsymbol{t}^w \in \mathbb{R}^{n_w}$. If one needs to predict derivatives such as velocity or acceleration of the observed profiles the interpolation should be smooth (e.g. a cubic spline). If no derivatives are needed, one should prefer simpler models such as linear interpolation as this reduces the nonlinear contribution of the derivative term in the linearization term Z and thus reduces the complexity the estimation problem, see model (**??**).

C: The covariance matrix of the random disparities $\boldsymbol{w}_i$ should be chosen to respect the experimental setup. If movements are modeled in percentual time, and the beginning and end of the observed trajectories correspond to the same states across movement (e.g. beginning and end of movement), the model for $\boldsymbol{w}_i$ should respect that. The simplest such model is to assume that $\boldsymbol{w}_i$ is a Brownian bridge observed at the discrete anchor points $\boldsymbol{t}^w$. For other types of data one may wish to include a random Gaussian time shift (add constant matrix to the covariance of the non-shift part of the model) or have an open end point which could be modeled using a Brownian motion model. If n_w is low relative to the number of repetitions one may model C as a completely free covariance matrix.

$\mathcal{S}$: A good allround choice for the covariance function of the amplitude variance is the Matérn covariance function. The Matérn covariance has three parameters, scale, range and smoothness. The scale parameter determines the variance of the process, the range parameter determines the strength of the correlation over time and the smoothness parameter determines the smoothness of the corresponding process. If one wants to simplify the optimization problem, one may fix the smoothness parameter at some value that represents sufficient smoothness, for example 2 corresponding to twice differentiable sample paths of the amplitude effect. For experiments with fixed start and end values one may use a bridge process such as the Brownian bridge, however, it is often preferable to use a less specific model than an overly specific model. For additive effects such as x_i, even slight misspecification of a bridge covariance structure at the beginning and end of movement (where variance and covariance go from zero to non-zero) may result in considerable bias of the corresponding parameters.

Consider the handwriting signature data in percentual time in Figure S1.The data consist of 12 acceleration magnitude profiles, each with 98 observations, corresponding to repetitions of a signature being written by a participant. This data has previously

been used as an example in [1]. A reproducible pattern across repetitions is in the nature of the task, and we also see a strong consistent pattern across the samples, but the curves are both misaligned and vary systematically in amplitude. Using the considerations above, we choose a B-spline basis with 40 interior knots to have sufficient flexibility to model the mean, we choose v to be a piecewise linear interpolation of the disparities $\boldsymbol{w}_i$ that we model as discretely observed Brownian bridges over $n_w = 20$ equidistant anchor points. For the amplitude covariance $\mathcal{S}$ we choose a Matérn covariance with unknown scale, range and smoothness.

The alignment of the proposed model is displayed in Figure S2.We see a neat alignment of the samples and a mean function that represents the mean pattern well, with no indications of local overfitting. Similarly for the warping functions, we only see small systematic deviations from the identity warp, despite of the high number of anchor points. The maximum likelihood estimates for the variance parameters were as follows: the warp scale estimate was 14.1, the variance scale estimate for the amplitude effect was 54.4, the range parameter estimate was $8.2 \cdot 10^{-3}$, the smoothness parameter was 6.2, and the noise variance σ^2 was estimated to be $1.4 \cdot 10^{-4}$. This suggests that the systematic part of the amplitude variation explains more than 99.9% of the amplitude variation, which fits well with the smooth functional samples.

To find the best among multiple models, one can compare different models using cross-vaildation, as was done in this paper, if such a setup is meaningful for the application at hand. In general one can do model selection based on the corrected conditional AIC of the linearized likelihoods [2].

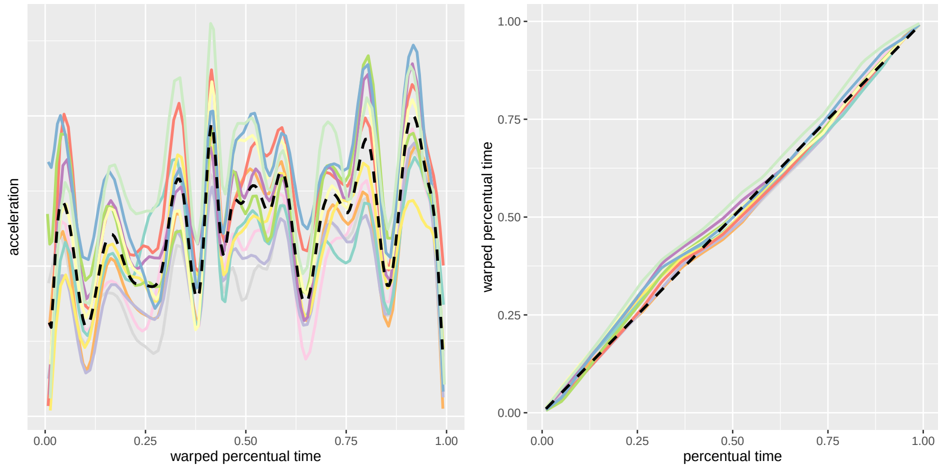

(a) Aligned samples and mean (dashed)

(b) Warping functions compared to the identity (dashed)

Figure S2. The aligned acceleration functions from Figure 1 (a), along with the predicted warping functions (b).

R code for fitting the model to the signature data

Suppose that `y` is a list containing the 12 acceleration trajectories and `t` is a list of the corresponding observation times. The model described above can be specified and fitted using the code given below. The methods in the `pavpop` R package are thoroughly documented with a wide array of examples in the package help pages and vignettes.

```
# Install and load pavpop R package
```

```
if (packageVersion("devtools") < 1.6) {
  install.packages("devtools")
}
devtools::install_github('larslau/pavpop')
library(pavpop)

# Set up basis function
kts <- seq(0, 1, length = 42)[2:41]
basis_fct <- make_basis_fct(kts = kts, intercept = TRUE,
                            control = list(boundary = c(0, 1)))

# Set up warp function
tw <- seq(0, 1, length = 20)
warp_fct <- make_warp_fct('piecewise-linear', tw)

# Set up covariance functions and roughly initialize parameters
warp_cov_par <- c(tau = 10)
warp_cov <- make_cov_fct(Brownian, noise = FALSE, param = warp_cov_par,
                         type = 'bridge')

amp_cov_par <- c(scale = 4, range = 1, smoothness = 2)
amp_cov <- make_cov_fct(Matern, noise = TRUE, param = amp_cov_par)

#
# Estimate in the model
#

# Rough bounds on parameters
lower <- c(1e-3, 1e-3, 1e-3, 1e-3)
upper <- c(1000, 10, 10, 10)

res <- pavpop(y, t, basis_fct, warp_fct, amp_cov, warp_cov,
              iter = c(5, 20), homeomorphism = 'soft',
              like_optim_control = list(lower = lower, upper = upper))
```

Simulation study

To evaluate the proposed algorithm for maximum likelihood estimation, we simulated data from the proposed model under the maximum likelihood estimates on the full data using a sampling setup identical to the central experiment ($d = 30.0$ cm, medium obstacle). We simulated 1000 outcomes and ran the estimation procedure as described in the section on modeling of effects and the algorithmic approach. The total runtime of the 1000 estimation procedures was approximately 6 hours on a 64-core machine.

The densities of the integrated square estimation errors (L^2 error) for the estimated mean profiles (experiment and participant) are shown in the right panel of Figure S3.For comparison, the experiment-specific and participant mean profiles have been estimated using ordinary least squares (OLS) estimation with the correctly specified spline model for the mean. The corresponding densities are shown in the left panel. We note that the densities are shown on squareroot scale to enable visual inspection of the differences. We see that the estimate for the experiment-specific mean profile is marginally more stable for the OLS estimation, but results are close-to perfect for both methods. For the participant-specific effects, however, we see that the proposed model that aligns samples within participant using a random warping

function gives L^2 errors that are approximately an order of magnitude lower than simple OLS estimation.

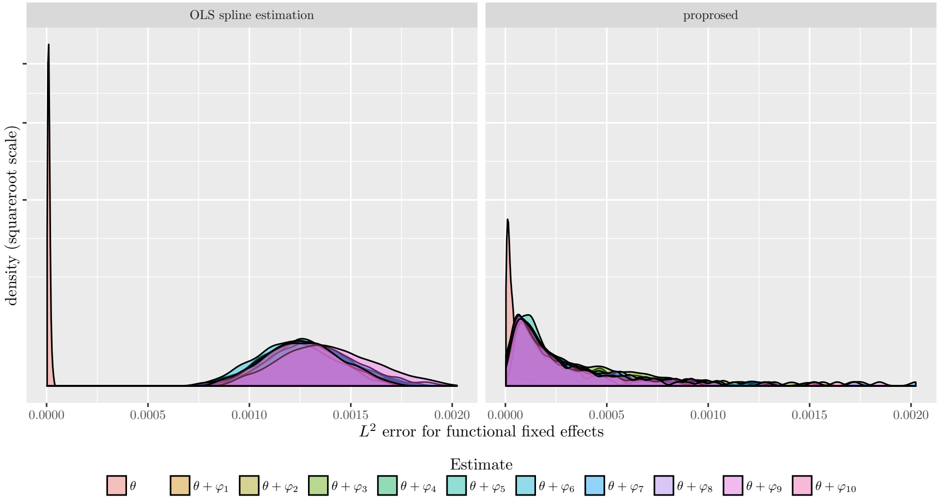

Figure S3. Densities of the integrated square estimation errors (L^2 errors) for the common and participant-specific mean functions in the simulation study. The left panel shows results for ordinary least square (OLS) estimation and the right panel shows the results for the proposed model and estimation algorithm. Both models were fitted using the correctly specified spline model for the mean. Note that the density is displayed on squareroot scale.

Figure S4 displays densities of the differences between the maximum likelihood estimates for the true participant-specific warping parameters across participants. The estimates generally seem unbiased with small variance around the true warping parameters.

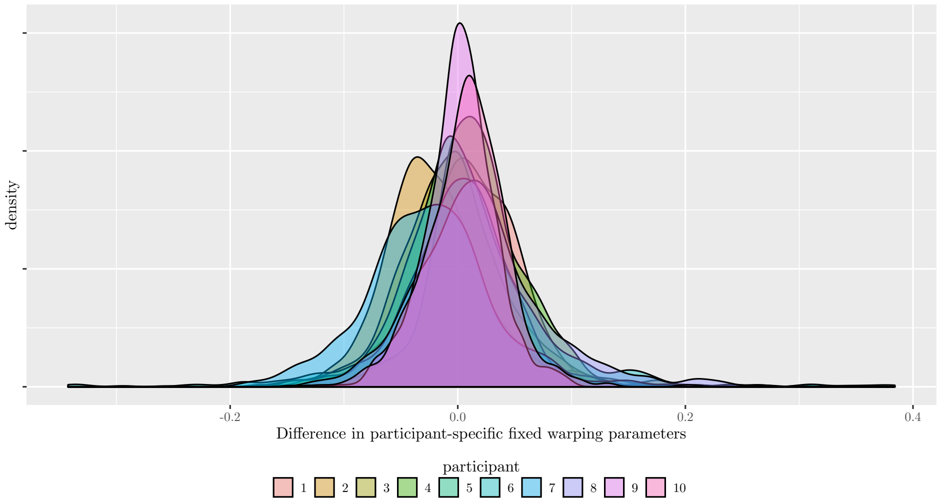

Figure S4. Densities of the difference between the estimated and the true participant-specific warping parameters across participants.

Figure S5 displays densities for the parameter estimates in the simulated experimental setups. We see that the estimators for the noise scale σ and the scales

for the warp parameters $\sigma\gamma$ both seem to have a small upward bias. The scale $\sigma\tau$ of the serially correlated effects and the range parameter $1/\alpha$ both seem to be estimated with very little or no bias. Slightly biased variance-parameter estimates are to be expected in likelihood-based inference [3], in particular in nonlinear models where bias-reducing estimation methods such as restricted maximum likelihood (REML), that are inherently linear, are not available.

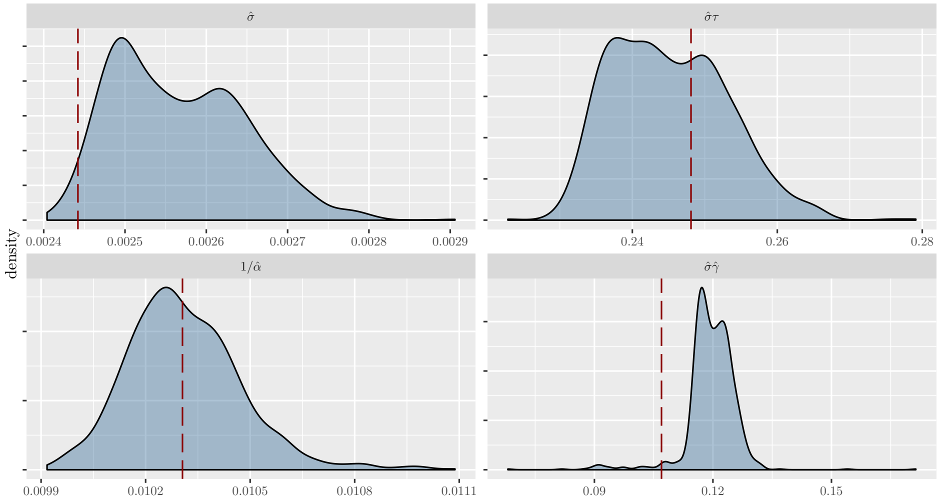

Figure S5. Densities of the estimated variance parameters in the simulated experimental setups. Dashed red lines indicate the true values of the parameters.

Cross-validation grids for motion classification

Cross-validation was done over:

MBM number of bands J in $\{1, 2, 3, 4, 5, 6\}$.

DTW degrees of freedom for B-spline basis $\{8, 13, 18, 23, 28, 33, 38\}$. For DTW_p $\{8, 9, \ldots, 18\}$.

FR number of principal components in $\{1, 2, 3, 4, 5\}$.

FR_E number of principal components in $\{1, 2, 3, 4, 5\}$ and weighting between phase and amplitude distance in $\{0.0, 0.5, \ldots, 5.0\}$.

References

1. Srivastava A, Wu W, Kurtek S, Klassen E, Marron J. Registration of functional data using Fisher-Rao metric. arXiv preprint arXiv:11033817. 2011;.

2. Greven S, Kneib T. On the behaviour of marginal and conditional AIC in linear mixed models. Biometrika. 2010;97(4):773–789.

3. Harville DA. Maximum likelihood approaches to variance component estimation and to related problems. Journal of the American Statistical Association. 1977;72:320–340.
